# Toxicity evaluation of ConvitVax breast cancer immunotherapy

**DOI:** 10.1038/s41598-021-91995-6

**Published:** 2021-06-16

**Authors:** María A. Duarte C., Jeismar M. Carballo O., Yetsenia M. De Gouveia, Angie García, Diana Ruiz, Teresa Gledhill, Eglys González-Marcano, Ana F. Convit

**Affiliations:** 1 Unidad Experimental de Inmunoterapia, Fundación Jacinto Convit, Caracas, Venezuela; 2 Jacinto Convit World Organization, Inc., Palo Alto, CA USA; 3 Facultad de Medicina, Universidad Central de Venezuela, Caracas, Venezuela; 4 Servicio de Anatomía Patológica, Hospital José María Vargas, Caracas, Venezuela

**Keywords:** Cancer, Immunology, Oncology

## Abstract

ConvitVax is a personalized vaccine for the treatment of breast cancer, composed of autologous tumor cells, bacillus Calmette-Guérin (BCG) and low concentrations of formalin. Previous pre-clinical studies show that this therapy induces a potent activation of the immune system and achieves an effective response against tumor cells, reducing the size of the tumor and decreasing the percentage of immunosuppressive cells. In the present study, we evaluate the toxicity of ConvitVax in healthy BALB/c mice to determine potential adverse effects related to the vaccine and each of its components. We used standard guidelines for pain, discomfort and distress recognition, continuously evaluated the site of the injection, and completed blood and urine clinical tests. Endpoint necropsy was performed, measuring the weight of organs and processing liver, kidney, thymus and lung for histological examination. Results show that the vaccine in its therapeutic dose, at 3 times its therapeutic concentration, and its individual components did not cause death or behavioral or biological changes, including any abnormalities in whole-body or organ weights, and tissue damage. These results support the safety of ConvitVax with minimal to no side-effects.

## Introduction

Breast cancer is the most common cancer in women worldwide and one of their main causes of death^1^. About 2.26 million new cases were reported in 2020 and it is expected that by the year 2030 new cases will increase by nearly 21% over the previous year^2^.

Approximately 80% of patients with breast cancer receive chemotherapy, hormonal therapy and/or radiotherapy, followed by tumor removal^3,4^. New treatments such as targeted therapies and immunotherapies are more recent strategies that are still in development^3,5^. Immunotherapy approaches aim to stimulate the patient's immune system and/or affect components of this system as a treatment (monoclonal antibodies, cytokines, among others)^6^. The most commonly used immunotherapies are vaccines and checkpoint inhibitors, such as the well-known anti-programmed death-ligand 1 (anti-PD-L1). However, the use of anti-PD-L1 has not been highly effective in breast cancer as a monotherapy, but in combination with standard neo-adjuvant or adjuvant therapies^7–9^. Moreover, recently published clinical trials have shown that immunotherapy has an important role in the treatment of this devastating condition^10–13^.

In 2006 Dr. Jacinto Convit proposed a breast cancer immunotherapy based on the combination of autologous tumor cells homogenate prepared with the patient’s own tumor tissue, combined with the bacillus Calmette-Guérin (BCG) vaccine, and low concentrations of formaldehyde (formalin)^14^. This therapy, now named ConvitVax, aims to stimulate the immune system of patients and achieve an effective and specific response against tumor cells^14,15^. Due to poor immunogenicity of tumor cells alone, many autologous tumor cells vaccine-based trials for breast cancer combine cells with additional antigens, cytokines or other immunomodulators^16^. BCG represents one of the most used adjuvants in immunotherapy regimens and is known to strongly activate the immune system against tumors. Moreover, a specific strain of BCG is FDA-approved for the treatment of superficial bladder cancer^17,18^. Also, the use of BCG in combination with other standard cancer treatments has shown to improve the therapeutic effect of the single agent^19–21^. These findings support the use of BCG as an adjuvant in ConvitVax for breast cancer treatment.

Our group has successfully used BCG as an immune adjuvant in the treatment of leprosy and leishmaniasis, conditions with specific immunological deficits at their core. In these studies, BCG was particularly effective in treating those diseases when combined with a dilute solution of formalin^22,23^. In the production process of vaccines, formalin is commonly used as a preservative though it has demonstrated that at low concentrations it induces an increase in the antigenic response, being also considered an adjuvant^24^. It should be noted that the concentration of formalin used in ConvitVax is very low, similar to that found in commercial vaccines such as the Polio^25^.

In previous studies with ConvitVax, we had conducted a tolerability analysis in animal models, showing apparent safety of the autologous vaccine^26^. Hence, a small human pilot study was performed in patients with advanced (mostly end-stage) breast cancer, obtaining positive and promising results with minimal side effects and a survival rate of 60% at a 5-year follow-up^26^. Later, to obtain mechanistic data of ConvitVax, a preclinical study was performed in BALB/c female mice with a 4T1 metastatic breast cancer model, where we observed a significant reduction of the tumor growth rate and an important infiltration of antitumor immune cells and plasma cells in the tumor microenvironment. The latter result suggested the possible establishment of immune system memory, which could help reduce disease recurrence^27^. In another study, ConvitVax was combined with anti-programmed cell death protein 1 (anti-PD-1) to further potentiate the anti-tumor effect of the vaccine, showing a slight improvement^28^. All these previous results demonstrated the likely effectiveness of ConvitVax against breast cancer, making it a potentially viable customized immunotherapy.

In past studies, a fixed concentration of ConvitVax’s components were used and evaluated, based on Dr. Convit’s vaccine proposal^14^. To continue the careful development of this therapy, here we conducted further controlled toxicity evaluations. The toxicity and immunogenicity of each component in ConvitVax and the combination itself were evaluated in healthy BALB/c female mice, a syngeneic mouse model, to determine any potential adverse effect that could be related to the treatment. To adequately compare with previous experimental studies, we used induced 4T1 tumors for the vaccine preparation. Also, a guideline for pain, discomfort and distress recognition was applied for this study based on the evaluation of weight loss, appearance, spontaneous behavior, behavior in response to manipulation and vital signs^29^. Local reaction at the site of the injection was evaluated at 4, 24 and 48 h after each dose, and a series of clinical laboratory tests were performed in blood and urine samples. Lastly, necropsies and histopathological examination of the main organs were performed.

## Materials and methods

### Tumor induction, cell line and preparation of ConvitVax

For tumor induction, the cell line 4T1 was used, since it specifically induces mammary-like tumors in immune intact BALB/c mice. This is an animal model for stage IV human breast cancer that has shown to closely recapitulate the immunogenicity, growth characteristics and metastatic properties of the human disease^30^. The 4T1 cells were cultured as indicated by the manufacturer and harvested as previously described^27^, to be further used for tumor induction. Primary tumors were induced in 10 BALB/c mice to obtain tumor tissue for the autologous tumor cells homogenate preparation. Specifically, 4T1 cells (1 × 10^6^ cell/mouse) were injected subcutaneously (s.c.) into the mammary fat pad of mice. When tumors reached a volume of 1.5–2.5 cm^3^, mice were anesthetized with excess Propofol (19 mg/Kg) injected intraperitoneal until unresponsive to toe tap and/or agonal breathing, and then euthanized to obtain the tumors.

Primary tumors were extracted in sterile conditions and stored in PBS plus penicillin–streptomycin 1 × (Sigma-Aldrich) at − 80 °C until used. Tumors were processed on the same day of vaccination following the protocol proposed by Convit et al.^26^ with minor modifications by Godoy et al.^27^. The vaccine mixture containing the appropriate concentrations of tumor cells homogenate, BCG, and formaldehyde (Table 1) were prepared in a final volume of 100 μL as previously described^26^, and administered immediately.

**Table 1:** Treatments administered for each group of mice.

Group	Treatments	N
1	Control (Vehicle-PBS)	9
2	Homogenate (0.6 mg)	9
3	(BCG—Danish strain 1331) (1.875 mg/ml)	6
4	Formalin (0.06%)	9
5	Homogenate (200 µg) + BCG (0.625 mg/ml) + Formalin (0.02%)	9
6	Homogenate (0.6 mg) + BCG (1.875 mg/ml) + Formalin (0.06%)	9

The 4T1 cell line was provided by the Cellular and Molecular Pathology Laboratory of Instituto Venezolano de Investigaciones Científicas (IVIC), maintained in the recommended medium and tested for mycoplasma before being used in the study. This study was approved by the “Comité de Bioética para la Investigación en Animales de Laboratorio de la Fundación Jacinto Convit” (CBIALFJC).

### Experimental animals

Since breast cancer prevalence is very low in males, we only utilized female mice in this study. Female BALB/c mice between 6 and 8 weeks were provided by Instituto de Estudios Avanzados (IDEA) and maintained in the animal research facility of Fundación Jacinto Convit (FJC) in computerized rodent housing connected to a Smartflow system (Tecniplast). The animals were under safe and monitored ventilation environment inside polysulfonate boxes, in separate units with ventilation systems, HEPA filtered air, controlled constant temperature of 25 °C, a relative humidity of 40–70% and a light cycle of 12 h light/12 h dark. They were provided open access to food and water and acclimated to the controlled environment for 7 days before starting the experiment. Animals were randomly divided in 6 boxes, one for each treatment group as specified in Table 1 and were identified using the method of ear punches. The treatment groups were defined as follows: Group 1 (G1) control group treated with PBS; Group 2 (G2) treated with high dose of autologous tumor cells homogenate (0.6 mg tumor cells homogenate); Group 3 (G3) treated with high dose of BCG (1.875 mg/ml); Group 4 (G4) treated with high dose of formalin (0.06%); Group 5 (G5) treated with the therapeutic concentration of ConvitVax (Homogenate (200 µg) + BCG (0.625 mg/ml) + Formalin (0.02%)), and Group 6 (G6) treated with 3 times the therapeutic concentration of ConvitVax (Homogenate (0.6 mg) + BCG (1.875 mg/ml) + Formalin (0.06%)) (Table 1).

### Study protocol overview

**Figure 1:**
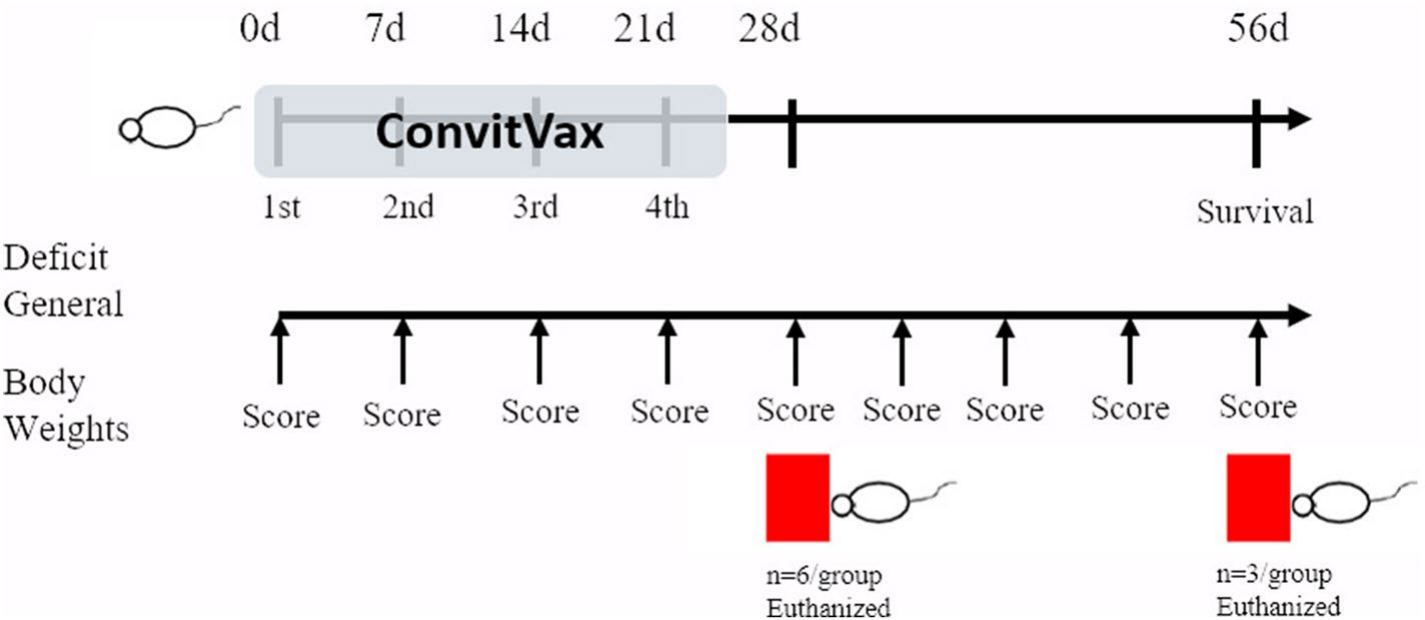
Timeline. The scheme represents the performed experimental protocol.

The study protocol is summarized in Fig. 1. Briefly, animals received their assigned treatments (Table 1) on day 0, 7, 14, and 21, followed by a 4-week recovery period.

### Toxicity study

51 female BALB/c mice between 6 and 8 weeks of age were randomly divided into the 6 groups indicated in Table 1. Since the BCG vaccine is widely used today as a tuberculosis vaccine and its adverse effects have been well studied^31–33^, following recommendations of the bioethics committee, we reduced the number of subjects in G3 to minimize the number of animals in the study. All mice received 100 μL of the corresponding treatment injected intradermal on the base of the neck once a week for 4 weeks, which was followed by a 4-week recovery period.

To determine any possible effects on the general condition of the animals, the parameters established by Morton and Griffiths^29^ described in the guideline for pain, discomfort and distress recognition were used. This guide is based on the evaluation of weight loss, appearance, spontaneous behavior, behavior in response to manipulation and vital signs. In addition to the characteristics described above, the endpoint of the study was achieved euthanizing the animals by injecting excess Propofol (19 mg/Kg) intraperitoneal to the treated animals^29,34^.

One week after finishing the treatment, on day 28, 6 mice were randomly taken from each experimental group, and euthanized. At this point any immediate or acute effect of ConvitVax could be potentially identified, while taking into account that in previous studies performed by Godoy et al.^27^, a clear immune activation induced by ConvitVax was determined 28 days after initiating treatment. The remaining mice from groups G1, G2, G4, G5 and G6, were kept in the study until day 56 to obtain the survival rate.

### Observation and animal’s behavior

**Figure 2:**
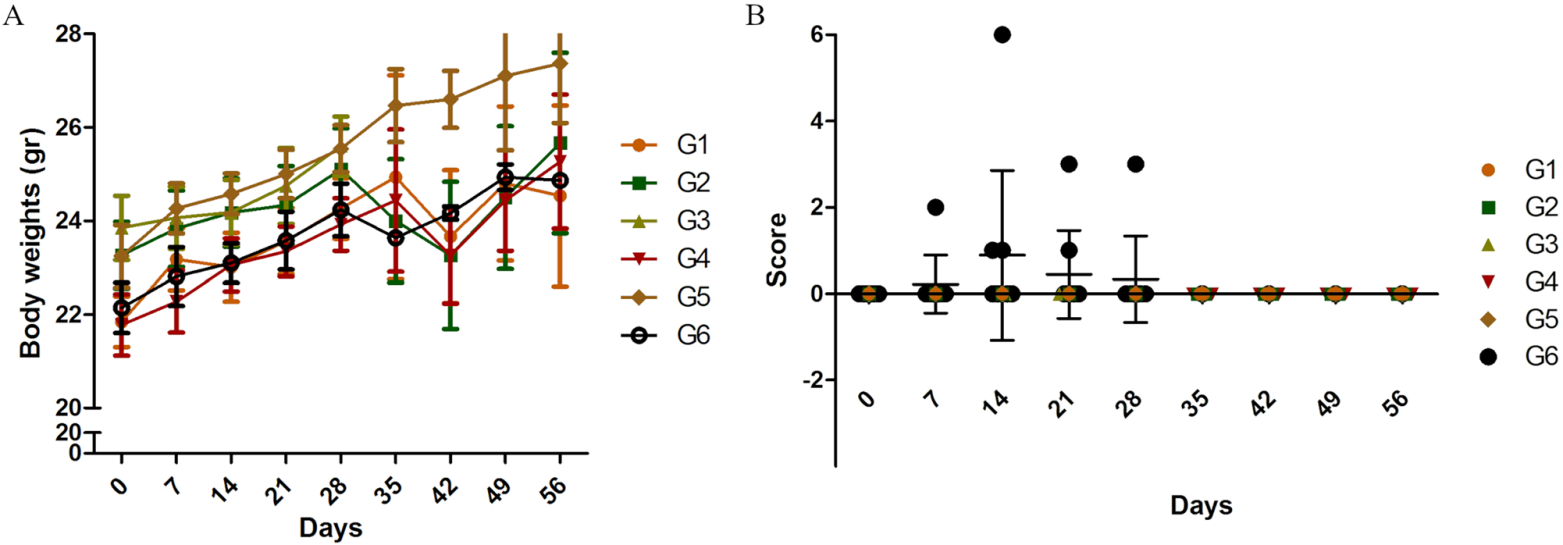
Body weights and general deficit. (**A**) Body weights determined in all study groups. (**B**) General deficit calculated in all study groups. The data is presented as the mean ± SEM of all mice in each group.

The animals were evaluated every 7 days, determining both their general deficit (GD) and weight in grams throughout the study (until 56 dpt) (Fig. 2). Weights were determined using an analytical balance (Xacta brand). The GD was determined using a scoring table described by Clark (1997), which briefly consists of the estimation of 6 characteristics: fur, ears, eyes, posture, spontaneous activity and epilepsy; obtaining a sum score between 0 and 28, considering 0 as normal and 28 as the highest GD^35^.

### Treatment effect at injection site

To evaluate the site of the injection, the area on the back of the neck was shaved prior to the application of the vaccine doses, allowing the visualization of local reactions to be determined for all groups. The site of the injection was evaluated at 4, 24 and 48 h after the application of the treatment, considering the following skin reactions: Normal (N), Papule (P), Erythema (E), Vesicle (V), Induration (I), Swelling (T), and Necrotic reaction (NR)^32^.

### Urine analytes

The analytes in the urine were determined at 28 dpt. The collection of urine was carried out using the method "urine collection without intervention (in mouse)" described by Kurien et al.^36^. In this protocol we used a one animal method, which allows a single mouse to urinate in a plastic wrap placed on white sheets of paper^36^. Bayer´s test strips were used for a semi-quantitative evaluation and also a microscopic analysis was performed to confirm the results of the strips^37^.

### Hematological and biochemical analysis

To determine the toxic effect of the treatments, 6 mice were extracted from each group at day 28, for a clinical evaluation with hematological and biochemical analyses. Mice were anesthetized with Propofol 19 mg/Kg intraperitoneal^34^. Once the lack of reflex was confirmed, the animal was exsanguinated by cardiac puncture for sampling. The blood samples were divided in two parts. One part was anticoagulated with 1% EDTA for hematological examination and the rest allowed to clot for biochemical analysis of the serum. Additionally, a smear was made from the whole blood sample. Hematological and biochemical analyses were performed by Laboratorio Corona, using the Landwind LWD 3600 (Insaide Lab C.A, USA) and ELAN Diagnostic ATAC8000 (Best Lab C.A., USA) equipment.

The euthanasia protocol applied to the experimental animals was done by injecting excess of anesthetic plus cervical dislocation.

### Necropsy

To evaluate the adverse effects of the treatments applied, necropsy and sampling of mice organs were carried out together with the clinical evaluation; 6 mice in each group were evaluated at 28 dpt.

Necropsy was performed after exsanguination of the animals. A primary incision was made at the abdominal level and then a secondary incision to open the thoracic cavity. Subsequently, the extraction of organs was performed individually (lungs, brain, heart, kidneys, liver, spleen, thymus, ovaries and uterus) and the individual net weight of each organ was recorded. In all cases the organs were weighed free of fat and connective tissue, avoiding any damage or artifact to the tissue^38^.

For further morphological and histopathological evaluation, the organs were preserved in 4% paraformaldehyde and immersed in a 30% sacarose suspension for at least 48 h, and then frozen by immersion in liquid nitrogen until use. Once the necropsy and sampling were finished, the waste material was discarded following biosafety precautions. All experimental procedures performed in the animals and described above were done in compliance with the rules for the use of research animals described in the code of ethics for life (Ministerio del Poder Popular para la Ciencia, Tecnología e Industrias Intermedias, 2011), and international guidelines such as "The ARRIVE guidelines", “USA Guidelines: Guide for the Care and Use of Laboratory Animals” and “EU Guidelines: Requirements for Establishments and for the Care and Accommodation of Animals”.

### Morphological evaluation of organs

To identify any morphological alteration related to the treatment, liver, kidney, thymus and lung were selected for further macroscopic evaluation at 28 dpt, recording images and describing all findings. This was carried out considering the following: general condition, color, borders, symmetry, consistency, and pathological findings.

### Histopathological analysis

The organs evaluated were sectioned using a scalpel and processed in a Leica CM1520 cryostat. The selected sections were placed in a cooling chamber at − 20 °C, embedded in cryogenic gel and cut to a thickness of 6–8 µm, with a total of 6 cuts per section. All cuts were fixed to microscope slides and then stained with Hematoxylin and Eosin (H&E).

For histopathological analysis, tissue samples were evaluated using the Leica DM2000 LED optical microscope linked to a digital camera CMOS with high definition through a Mosaic software. A minimum of 4 tissue samples per individual were processed. 10 optical fields were visualized with 10X, 20X, and 40X objectives, and all findings adequately recorded and analyzed.

For this evaluation, the criteria considered were the morphology of the cells and tissues, as well as any alteration observed in the cellular responses within each tissue, such as hyperplasia, hypertrophy, edema, necrosis, apoptosis and inflammation.

### Statistical analysis

The results obtained were expressed as mean ± standard error mean (± SEM). The means were calculated based on the individual values of each animal. Statistical analysis of data was performed using ANOVA followed by Dunnett’s test for comparisons of differences between group mean values. The level of statistics significance for all tests were p < 0.05 level.

## Results

### General symptoms, survival and body weights

The general condition of the animals was evaluated following the guidelines described by Morton and Griffiths^29^, with all groups having normal behavior, with no apparent symptoms of toxicity at any of the observed times of the study. Additionally, no death occurred during the treatment or the post necropsy recovery period in the remaining animals, with a 100% survival rate. The follow up on each animal’s body weight showed normal development, as there were no significant differences in the mean body weights between the animals in the treated groups and those in the control group (Fig. 2A). At day 42, a slight drop in weight was recorded for some groups, corresponding to about 5% diminution compared to the previous day. However, these changes in the treated groups were not statistically significant. No other recognizable change was observed in the animals that could be related to this apparent weight loss; also, at day 49, body weight was slightly recovered. Therefore, we cannot relate this event to the treatment or its time of application.

### General deficit status of mice

The general condition of the mice was evaluated as described by Clark^35^. The results are shown in Fig. 2B as individual dots for each mouse evaluated, with no difference between groups G1, G2, G3, G4 and G5. Only in G6, 3 mice showed minimal alterations and one animal a hyper-excitation event. These differences were only identified as statistically significant at 14 dpt (p < 0.001) (G1) (Fig. 2B), with the highest GD score observed being 6. During the rest of the experiment and after 28 dpt, all mice evaluated showed scores of 0.

### Treatment effect at injection site

**Figure 3:**
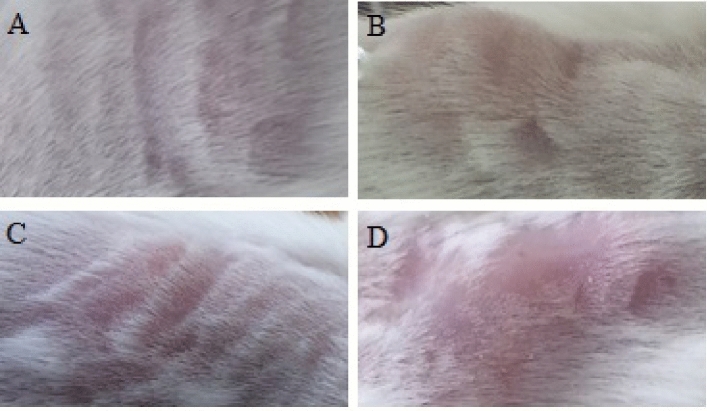
Reactions observed in the site of injection. (**A**) Normal skin; (**B**) Papule; (**C**) Erythema; (**D**) Papule plus erythema.

To determine possible local adverse effects of the different treatments, the injection site was regularly observed and evaluated in all groups. Animals receiving the vaccine (G5 and G6) and BCG alone (G3) showed a mild and localized reaction mainly with formation of a papule and in some cases presence of erythema (Fig. 3). In all cases the reaction observed was as expected, and it resolved spontaneously before the endpoint of the study (lasting no more than 2 weeks after treatment), with no further reaction during the next days until the endpoint of the experiment.

### Urine analytes

The urinalyses were performed after treatment, and were all normal. Namely, there were no leukocytes, nitrites, protein, glucose, ketone bodies, bilirubin, urobilinogen and erythrocytes identified, and density and pH values were in the normal range. The urinalysis results obtained at 28 dpt are summarized in Table 2, all showing no significant differences (p > 0.05) relative to the control group.

**Table 2:** Urine analytes in mice treated with ConvitVax or its components in repeated doses.

Time and groups	Day 28					
	G1	G2	G3	G4	G5	G6
n	6	6	6	6	6	6
Density	1025.0 ± 0.0	1025.0 ± 0.0	1025.8 ± 2,0	1025.8 ± 2.0	1026.7 ± 2.6	1025.0 ± 4.5
pH	5.5 ± 0.6	5.5 ± 0.6	5.2 ± 0.4	5.3 ± 0.5	5.3 ± 0.5	5.8 ± 0.4
LEU	n	n	n	n	n	n
NIT	n	n	n	n	n	n
PRO	n	n	n	n	n	n
GLU	n	n	n	n	n	n
KET	n	n	n	n	n	n
BILI	n	n	n	n	n	n
URO	n	n	n	n	n	n
ERI	n	n	n	n	n	n

The data is presented as the mean ± SEM of all mice in each group.

Abbreviations: *LEU* leukocytes, *NIT* nitrites, *PRO* protein, *GLU* glucose, *KET* ketone bodies, *BILI* bilirubin, *URO* urobilinogen, *ERI* erythrocytes, *n* negative.

### Hematology and biochemical profiles

The potential toxic effects of treatment at day 28 as determined by hematological and biochemical profiles are summarized in Tables 3 and 4. Hematological analyses showed that G6 (High dose group) resulted in a non-significant increase in overall white blood cell count (WBC) and in polymorphonuclear leukocyte (PMN) count compared to control (G1). Additionally, G6 showed slight decreases in mononuclear leukocytes count (MN), hemoglobin (HGB) and hematocrit (HCT) (Table 3). Other than the PMN increase, the other non-significant changes relative to control were not considered to be biologically significant (Table 3). The biochemical profile analysis in all groups showed no significant changes in Creatinine (CR), Alkaline Phosphatase (ALP) and Sodium (Na +) (Table 4). Therefore, no unanticipated changes that could have been induced by the treatments were identified.

**Table 3:** Hematologic analysis in mice treated with ConvitVax or its components in repeated doses.

Time and groups	Day 28					
	G1	G2	G3	G4	G5	G6
n	6	6	6	5	6	6
RBC(× 10^12^/μL)	4.8 ± 0.2	4.6 ± 1.0	4.6 ± 0.8	5.2 ± 0.6	5.1 ± 0.7	4.3 ± 0.6
HGB (g/dL)	13.6 ± 0.9	13.3 ± 2.7	13.3 ± 2.2	15.1 ± 1.6	14.7 ± 2.5	12.5 ± 1.6
HCT (g/dL)	43.6 ± 2.0	41.4 ± 8.6	42.0 ± 7.0	47.1 ± 5.1	46.4 ± 5.9	39.2 ± 5.6
MCV(fL)	83.6 ± 3.6	91.5 ± 22.8	88.9 ± 16.4	78.0 ± 9.1	79.7 ± 11.9	94.5 ± 14.4
MCH (pg)	28.4 ± 0.7	29.3 ± 0.7	28.9 ± 0.2	29.2 ± 0.4	28.6 ± 1.4	29.1 ± 0.7
MCHC (%)	31.3 ± 0.8	32.2 ± 0.7	31.8 ± 0.3	32.1 ± 0.5	31.5 ± 1.5	31.9 ± 0.7
PLT (× 10^9^/L)	368.5 ± 56.6	393.0 ± 53.7	409.7 ± 54.6	407.4 ± 47.2	376.5 ± 67.5	379.5 ± 51.9
WBC (× 10^9^/L)	8.8 ± 2.0	9.0 ± 1.3	10.5 ± 0.5	9.5 ± 1.8	9.1 ± 1.9	10.8 ± 1.0
PMN (%)	26.8 ± 9.6	37.0 ± 12.4	33.8 ± 9.2	36.6 ± 7.8	35.0 ± 5.9	45.0 ± 17.3
MN (%)	73.2 ± 9.6	63.0 ± 12.4	66.2 ± 9.2	63.4 ± 7.8	65.0 ± 5.9	61.3 ± 9.1

It is important to mention that due to the difficulty of the exsanguination procedure, in some cases an insufficient amount of sample was obtained for the complete analysis. Therefore, the number of mice was variable and is shown in the table. The data is presented as the mean ± SD of all mice in each group.

Abbreviations: *RBC* red blood cell count, *HGB* hemoglobin concentration, *HCT* hematocrit, *MCV* mean corpuscular volume, *MCH* mean cell hemoglobin, *MCHC* mean cell hemoglobin concentration, *PLT* platelets, *WBC* white blood cell count, *PMN* polymorphonuclear leukocyte, *MN* mononuclear leukocytes.

**Table 4:** Clinical chemistry analysis in mice treated with ConvitVax or its components in repeated doses.

Time and groups	Day 28					
	G1	G2	G3	G4	G5	G6
n	**6**	5	4	6	6	4
CR (mg/dL)	**1.1 ± 0.6**	1.1 ± 0.3	1.5 ± 1.0	1.3 ± 0.6	1.6 ± 1.1	0.9 ± 0.5
ALP (U/L)	**103.3 ± 7.0**	103.8 ± 7.4	97.6 ± 4.4	116.8 ± 41.5	113.5 ± 17.1	114.0 ± 15.0
Na + (U/L)	**139.4 ± 1.8**	139.5 ± 0.7	140.2 ± 1.5	138.6 ± 1.9	139.2 ± 1.2	138.8 ± 0.5

It is important to mention that due to the difficulty of the exsanguination procedure, in some cases an insufficient amount of sample was obtained for the complete analysis. Therefore, the number of mice was variable and is shown in the table.

Abbreviations: *CR* creatinine, *ALP* alkaline phosphatase, *Na*^*+*^ sodium.

### Necropsy

Individual absolute organ weights (gr) were determined after the necropsy procedure on either day 28 or the endpoint of the study (day 56). This is considered an indirect measure of the general effect of the different treatments in mice. This data is presented in the Supplementary Table (S1). The results at day 28 indicated an increase in the weight of the lungs (in G4) and thymus (in G2) when compared to the control group (Supplementary Table S1-A).

To adjust for differences in overall body weight on the organ weights, we used the ratio of the organ weight to the whole-body weight (Supplementary Table S1-B) and organ weight to the brain weight (Supplementary Table S1-C). The latter represents a surrogate measure for lean body mass, which is not usually affected by treatments^39^. In this study we only observed a significant variation in the ratios of the organ’s weight for the thymus-to-brain ratio in group G2 (Homogenate High-dose) (Supplementary Table S1-B). Since the main changes observed in the treated groups were not significantly different from the control group, we cannot directly relate them to a treatment effect or its time of application. Therefore, these changes in organ weight are not considered biologically significant (Supplementary Table S1-A, S1-C).

### Histopathological findings

Macroscopic evaluation of the liver, kidney, thymus and lungs showed normal consistency, defined edges and adequate color in all the groups evaluated when compared with the controls. Foci of hemorrhage or necrosis were not observed.

**Figure 4:**
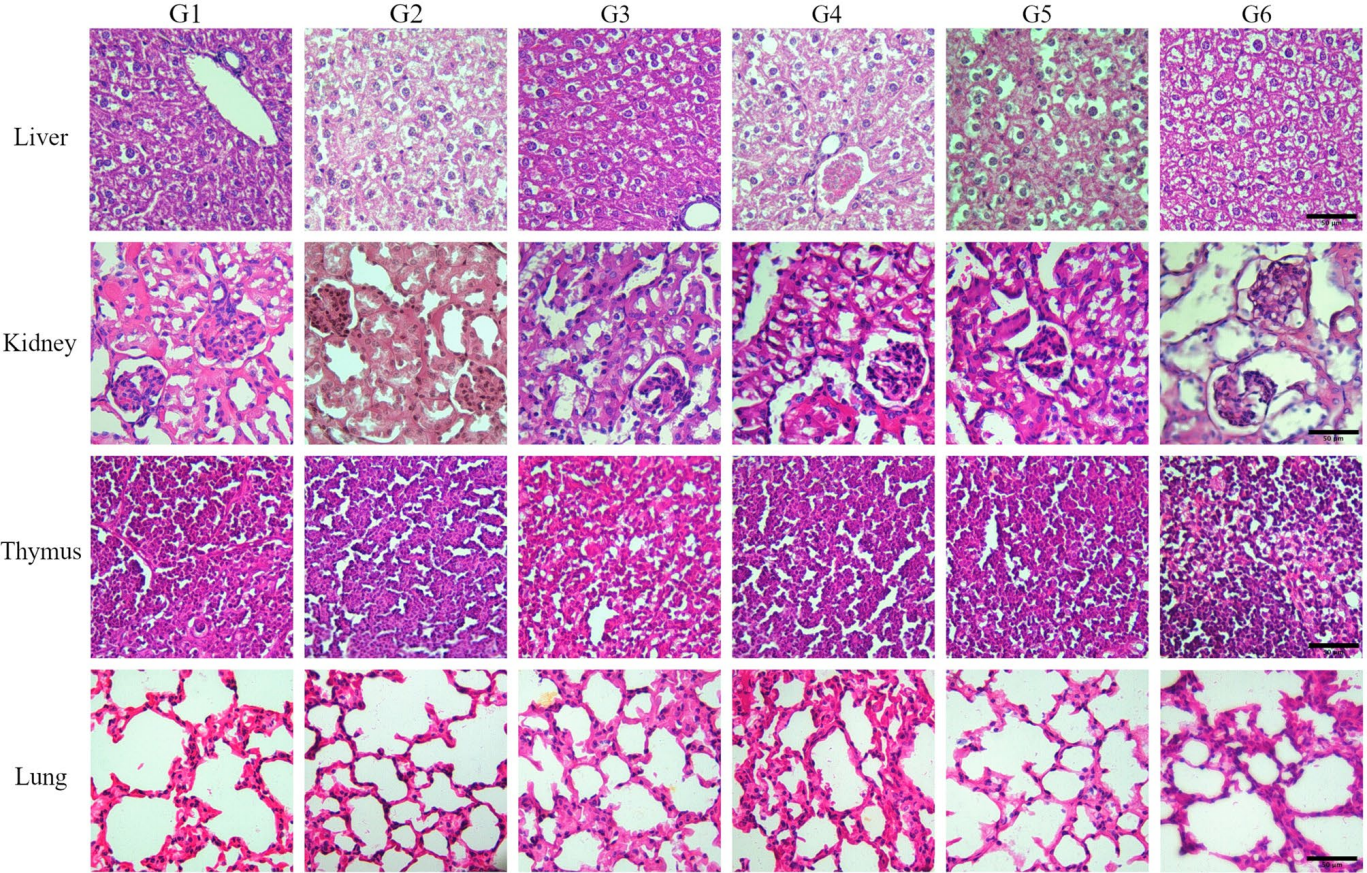
Histopathological analysis of different organ tissues from mice treated with ConvitVax. Liver, kidney, lung and thymus slides were processed and stained with H&E. Images shown are with 40X objective. Scale bar 50 µm.

Microscopic examinations with H&E staining showed that ConvitVax had no effect on the organs studied (Fig. 4). All findings were consistent with normal background lesions for age in strain-matched clinically normal mice. The changes were considered spontaneous and/or incidental in nature and unrelated to the treatment.

The liver sections of all treated groups showed preserved lobular and trabecular architecture, undamaged hepatocytes, occasionally binucleated with abundant eosinophilic cytoplasm. No apoptotic bodies nor inflammatory cells were observed. The portal tracts and the liver sinusoids were morphologically intact (Fig. 4).

The texture of glomeruli and renal tubules had normal renal cortex and glomeruli, glomerular capillary wall of regular thickness and normal basement membrane, normal mesangial and extracellular matrix, and Bowman spaces free and conserved, in both treated and control animals. No edema, fibrosis or inflammatory infiltrate were observed in the interstitium. Proximal and distal tubules, and collecting ducts were lined by an intact epithelium and basement membrane, and no crystals nor inflammatory cells were observed. Kidney arteries and arterioles showed intact endothelium and normal thickness (Fig. 4).

Thymus tissue sections analysis also revealed a normal pattern, with preserved thymic architecture, poorly defined corticomedullary boundaries and contours. No fibrosis, necrosis or atrophy were observed in the control or treated groups (Fig. 4).

The lung sections of all treated groups when compared with the control group, showed an unchanged pulmonary parenchyma. The airways were lined by typical epithelium, and the different cell types were identified. Open alveolar spaces with occasional foci of intraalveolar proteinaceous material was observed. No hyaline membranes were noted. Alveolar septa showed regular thickness. Pulmonary vessel showed intact endothelium. No fibrosis nor inflammatory cells were found. In addition, no fibrosis, necrosis, atrophy or inflammatory infiltrate were seen in any of the evaluated organs of the 6 groups studied (Fig. 4).

## Discussion

To confirm the safety of ConvitVax in a murine model we conducted a repeat-dose toxicity study in female BALB/c mice. All mice received 100 μL of the corresponding treatment (Table 1) injected intradermal on the base of the neck once a week for 4 weeks, followed by a 4-week recovery period. The aim of the study was to evaluate the toxicity of ConvitVax itself in its therapeutic concentration, at 3-times higher dose, as well as its components separately also at 3-times higher concentration. For the homogenate of tumor cells, it was important to confirm that the procedure was safe and that its inoculation did not induce a local or systemic proliferation of the tumor cells. We observed that in all groups receiving tumor cell homogenate (G2, G5 and G6) none of the mice developed a tumor of any kind and no tumor cell proliferation in the inoculated area or in any organ. Additionally, all animals remained in optimal general condition, indicating that the vaccine as prepared did not induce morbidity or cause cancer in the evaluated time.

BCG has well-known adverse effects during its clinical application. These include local swelling, infiltration, suppuration, ulcers, occasional inflamed lymph nodes, drug-induced lupus, scar formation at the site of inoculation and arthritis^40,41^. To ascertain induction of unexpected side effects, we carried out a regular observation of the injection site in all groups. As expected among immune-competent subjects, groups treated with ConvitVax or BCG alone showed a mild localized reaction (papule and erythema) that resolved spontaneously in no more than 2 weeks after treatment. There were no significant or potentiated adverse effects when applying the vaccine. This result indicates that the application of ConvitVax or BCG alone in the concentrations used, do not induce a significant adverse effect or toxic reaction, beyond that expected for BCG.

Another component of ConvitVax is formalin, which is a chemical compound used commonly in vaccine production to inactivate viruses, such as Hepatitis A, Polio, Influenza, among others^42,43^. Although its use remains controversial due mostly to allergic reaction to it, many vaccines currently used, contain very low concentrations of formalin, which remain in the final product after the development process^25,42^. The concentration of formalin used in the production of ConvitVax is similar to that found in many commercial vaccines such as the Polio, which also contains 0.02% formalin. The use of this compound in the production of vaccines has been related to its utility as a preservative. It is also considered an adjuvant since its use in low concentrations has shown to induce an increase in the antigenic response^24^. Likewise, low concentrations of formalin, although denaturing proteins, help conserve oligosaccharide epitopes, which are important in a specific immunological response^25^. This is accomplished through oligosaccharides recognition by APCs, increasing antigen uptake and activating several intracellular signaling pathways, resulting in cytokine secretion, cell activation, phagocytosis and antigen presentation that leads to the differentiation of CD4^+^ T cells and the activation of adaptive immune responses^44^.

Given its utility as a preservative and its ability to increase the antigenic response, this low concentration of formalin was included in the protocol for ConvitVax. In previous studies we demonstrated an improved effect when adding formalin relative to the use of autologous cells (homogenate) plus BCG alone, indicating that all three components are necessary to induce a potentiated antitumor effect^27^. In this study, we did not see any toxic effects in the treated mice that could be assigned to 0.02% formalin, the indicated concentrations.

In the present study no obvious signs of systemic toxicity or abnormalities in whole-body weight were observed. None of the mice evaluated lost more than 5% body weight and more importantly no significant changes were recorded in the organ to whole-body weight ratio. Organ weight can be a very sensitive indicator of an adverse effect of an experimental treatment^39^, thus our results suggest the lack of toxic effect induced by the treatments used in this study. Furthermore, during the time of the study, no deaths occurred and none of the mice developed a deteriorated condition. Additionally, most mice presented a GD score within normal range; only in group G6 did we observe a significant difference in the GD score at day 14, where 3 mice showed higher activation and one exhibited hyper-excited behavior, which were not maintained over time. Although the weight and overall structure of the animals' brains in all groups were normal, some investigators have suggested that these behaviors may be associated with neurological involvement and may be related to the presence of formalin^45^. No pathological changes were determined in the urine analyses as well as no abnormalities were identified in the necropsy and histopathological examination relative to control animals. Histopathological analyses of multiple organs revealed that there were no detectable variations in the normal architecture of tissues, allowing us to conclude that the treatments used did not affect the functioning of organs. When evaluating toxic effects of drugs, hematological analyses have a high predictive value for risk assessment, as the hematopoietic system is one of the most sensitive targets for toxic chemicals. In this study, we found no significant changes in the hematological parameters evaluated, whereby all the values determined were within the normal range, indicating that ConvitVax and its components had no toxic effect directed to the hematopoietic system.

The results presented here indicate that the use of ConvitVax in the indicated dosage, time schedule and described protocol does not produce any evidence of toxicity in mice. This also applies to the longer period of observation (56 dpt), during which urinalysis, hematology and pathological analyzes showed normal values and normal structure of the tissues (data not shown). These observations confirm the results observed at 28 dpt. In concordance with previous studies, where no apparent adverse effects were observed, here we show and are able to conclude that ConvitVax and its components at the indicated concentrations are non-toxic. Our data further suggest that the preparation method of our vaccine is likely safe and does not show an increased risk of tumor cell proliferation. Based on this evaluation and previous data published, we can reiterate that within the protocol used, ConvitVax has a safety profile, meriting its further development and advance into a phase I clinical trial to confirm the established therapeutic concentration for potential patient applications.

## Supplementary Information

Supplementary Information.

## Data Availability

The datasets built and analyzed in the present study are available from the corresponding author upon request.
