# Supplementary material for: Toxicity evaluation of ConvitVax breast cancer immunotherapy
Source: Sci Rep. 2021 Jun 16;11:12669. doi: 10.1038/s41598-021-91995-6 (PMC8209199; doi:10.1038/s41598-021-91995-6)
Supplement: Supplementary file 1 — Supplementary Information. [file 41598_2021_91995_MOESM1_ESM.pdf]

Supplementary Information

Supplementary Table S1. Organ weights, the ratio of organ to whole-body and the ratio of organ to brain weight in mice treated with ConvitVax or its components in repeated doses.

Time and groups	Day 28						Day 56				
	G1	G2	G3	G4	G5	G6	G1	G2	G4	G5	G6
n	6	6	6	6	6	6	3	3	3	3	3
(A)											
Brain (g)	0.48 ± 0.02	0.45 ± 0.02	0.49 ± 0.03	0.56 ± 0.16	0.48 ± 0.02	0.49 ± 0.03	0.43 ± 0.06	0.44 ± 0.06	0.49 ± 0.03	0.51 ± 0.03	0.49 ± 0.03
Heart	0.12 ± 0.01	0.11 ± 0.01	0.13 ± 0.02	0.20 ± 0.13	0.13 ± 0.02	0.11 ± 0.01	0.12 ± 0.02	0.11 ± 0.03	0.12 ± 0.01	0.12 ± 0.01	0.08 ± 0.04
Liver	1.33 ± 0.04	1.27 ± 0.13	1.27 ± 0.18	1.32 ± 0.16	1.30 ± 0.12	1.35 ± 0.16	1.24 ± 0.14	1.27 ± 0.23	1.30 ± 0.02	1.48 ± 0.17	1.29 ± 0.04
Spleen	0.11 ± 0.02	0.10 ± 0.03	0.19 ± 0.02	0.10 ± 0.02	0.11 ± 0.01	0.12 ± 0.01	0.10 ± 0.02	0.10 ± 0.03	0.11 ± 0.01	0.11 ± 0.01	0.09 ± 0.02
Lung	0.17 ± 0.01	0.17 ± 0.02	0.16 ± 0.01	0.26 ± 0.14*	0.18 ± 0.02	0.19 ± 0.03	0.18 ± 0.02	0.19 ± 0.03	0.18 ± 0.05	0.22 ± 0.06	0.23 ± 0.06
Thymus	0.05 ± 0.01	0.08 ± 0.02*	0.06 ± 0.03	0.04 ± 0.01	0.04 ± 0.01	0.05 ± 0.02	0.04 ± 0.01	0.04 ± 0.01	0.04 ± 0.00	0.06 ± 0.03	0.07 ± 0.01
Kidney	0.30 ± 0.02	0.27 ± 0.04	0.29 ± 0.06	0.39 ± 0.16	0.30 ± 0.25	0.30 ± 0.04	0.30 ± 0.05	0.27 ± 0.05	0.33 ± 0.02	0.33 ± 0.04	0.29 ± 0.07
Reproductive organs	0.20 ± 0.11	0.23 ± 0.05	0.16 ± 0.08	0.29 ± 0.13	0.19 ± 0.08	0.22 ± 0.08	0.14 ± 0.06	0.16 ± 0.08	0.23 ± 0.08	0.14 ± 0.03	0.17 ± 0.10

(B)

Brain	1.98 ± 0.14	1.84 ± 0.10	1.99 ± 0.19	2.27 ± 0.66	1.95 ± 0.15	1.99 ± 0.16	1.84 ± 0.28	1.89 ± 0.43	2.11 ± 0.42	2.18 ± 0.34	2.09 ± 0.30
Heart	0.48 ± 0.04	0.46 ± 0.05	0.51 ± 0.09	0.84 ± 0.58	0.51 ± 0.09	0.47 ± 0.05	0.49 ± 0.02	0.49 ± 0.19	0.50 ± 0.11	0.52 ± 0.11	0.35 ± 0.19
Liver	5.38 ± 0.17	5.18 ± 0.51	5.21 ± 0.91	5.40 ± 0.66	5.31 ± 0.69	5.49 ± 0.57	5.24 ± 0.39	5.48 ± 1.71	5.54 ± 0.83	6.25 ± 0.38	5.48 ± 0.87
Spleen	0.46 ± 0.07	0.40 ± 0.10	0.41 ± 0.10	0.42 ± 0.10	0.44 ± 0.06	0.47 ± 0.04	0.43 ± 0.02	0.42 ± 0.17	0.47 ± 0.11	0.48 ± 0.08	0.41 ± 0.13
Lung	0.68 ± 0.07	0.71 ± 0.08	0.67 ± 0.09	1.07 ± 0.59	0.73 ± 0.11	0.76 ± 0.10	0.77 ± 0.05	0.81 ± 0.22	0.78 ± 0.13	0.94 ± 0.26	1.01 ± 0.36

Thymus	0.19 ± 0.05	0.33 ± 0.10	0.23 ± 0.11	0.17 ± 0.02	0.16 ± 0.03	0.22 ± 0.08	0.17 ± 0.06	0.15 ± 0.05	0.17 ± 0.02	0.26 ± 0.08	0.29 ± 0.02
Kidney	1.24 ± 0.10	1.09 ± 0.19	1.21 ± 0.25	1.58 ± 0.66	1.24 ± 0.13	1.21 ± 0.16	1.26 ± 0.06	1.18 ± 0.37	1.37 ± 0.10	1.38 ± 0.19	1.22 ± 0.26
Reproductive organs	0.82 ± 0.48	0.92 ± 0.19	0.63 ± 0.30	1.19 ± 0.55	0.76 ± 0.34	0.90 ± 0.34	0.60 ± 0.22	0.66 ± 0.34	0.97 ± 0.38	0.59 ± 0.19	0.70 ± 0.32

(C)

Heart	24.17 ± 2.63	24.99 ± 2.72	25.73 ± 2.11	33.63 ± 13.52	33.63 ± 13.52	23.83 ± 4.27	26.94 ± 5.374	25.69 ± 5.46	23.71 ± 2.14	23.94 ± 2.09	16.64 ± 8.57
Liver	272.90 ± 13.48	280.60 ± 23.84	260.40 ± 23.70	247.00 ± 42.35	247.00 ± 42.35	278.70 ± 41.66	290.50 ± 4.28	289.60 ± 2.37	265.00 ± 3.20	291.10± 35.22	262.30 ± 9.41
Spleen	23.29 ± 3.03	21.48 ± 4.73	20.23 ± 3.23	19.16 ± 5.05	19.16 ± 5.05	23.97 ± 2.53	24.02 ± 5.10	21.51 ± 4.74	22.36 ± 1.80	21.97 ± 0.70	19.12 ± 3.43
Lung	34.17 ± 1.87	38.15 ± 3.15	33.59 ± 2.85	44.95 ± 13.73	44.95 ± 13.73	38.57 ± 7.25	42.60 ± 8.14	43.39 ± 11.71	37.42 ± 1.45	43.42 ± 10.43	47.43 ± 11.01
Thymus	9.38 ± 2.61	17.50 ± 5.03*	11.84 ± 5.52	8.09 ± 2.62	8.09 ± 2.62	11.27 ± 4.16	9.39 ± 3.04	8.17 ± 3.82	8.38 ± 1.07	12.50 ± 6.27	14.10 ± 1.34
Kidney	62.78 ± 3.91	59.09 ± 9.15	60.20 ± 8.15	67.96 ± 9.18	67.96 ± 9.18	61.07 ± 7.23	69.68 ± 12.54	61.90 ± 8.04	66.13 ± 8.47	63.91 ± 6.57	58.53 ± 12.55
Reproductive organs	41.49 ± 24.04	49.85 ± 9.61	32.33 ± 16.91	51.22 ± 17.02	51.22 ± 17.02	45.98 ± 19.03	32.01 ± 8.21	34.04 ± 15.75	45.54 ± 16.10	26.65 ± 5.20	34.29 ± 17.92

Note: The data is presented as the mean ± SD of all mice in each group.* Indicates significant differences at $p<0.05$ level, when compared with the control group. (A) Organ weights in mice treated with ConvitVax or its components in repeated doses (B) The ratio of organ to whole-body weight in mice treated with ConvitVax or its components in repeated doses and (C) The ratio of organ to brain weight in mice treated with ConvitVax or its components in repeated doses.
